# Investigation of the interactions of critical scale-up parameters (pH, *p*O_2_ and *p*CO_2_) on CHO batch performance and critical quality attributes

**DOI:** 10.1007/s00449-016-1693-7

**Published:** 2016-10-17

**Authors:** Matthias Brunner, Jens Fricke, Paul Kroll, Christoph Herwig

**Affiliations:** 1 Research Division Biochemical Engineering, Vienna University of Technology, Gumpendorferstrasse 1a 166/4, Vienna, 1060 Austria; 2 CD Laboratory on Mechanistic and Physiological Methods for Improved Bioprocesses, Vienna University of Technology, Gumpendorferstrasse 1a/166, 1060 Vienna, Austria

**Keywords:** CHO cell culture, Process parameter, Scale-up, Monoclonal antibody CQA, Design of experiments

## Abstract

Understanding process parameter interactions and their effects on mammalian cell cultivations is an essential requirement for robust process scale-up. Furthermore, knowledge of the relationship between the process parameters and the product critical quality attributes (CQAs) is necessary to satisfy quality by design guidelines. So far, mainly the effect of single parameters on CQAs was investigated. Here, we present a comprehensive study to investigate the interactions of scale-up relevant parameters as pH, *p*O_2_ and *p*CO_2_ on CHO cell physiology, process performance and CQAs, which was based on design of experiments and extended product quality analytics. The study used a novel control strategy in which process parameters were decoupled from each other, and thus allowed their individual control at defined set points. Besides having identified the impact of single parameters on process performance and product quality, further significant interaction effects of process parameters on specific cell growth, specific productivity and amino acid metabolism could be derived using this method. Concerning single parameter effects, several monoclonal antibody (mAb) charge variants were affected by process *p*CO_2_ and pH. *N*-glycosylation analysis showed positive correlations between mAb sialylation and high pH values as well as a relationship between high mannose variants and process pH. This study additionally revealed several interaction effects as process pH and *p*CO_2_ interactions on mAb charge variants and *N*-glycosylation pattern. Hence, through our process control strategy and multivariate investigation, novel significant process parameter interactions and single effects were identified which have to be taken into account especially for process scale-up.

**Electronic supplementary material:**

The online version of this article (doi:10.1007/s00449-016-1693-7) contains supplementary material, which is available to authorized users.

## Introduction

Monoclonal antibodies (mAbs) have become the main products of biopharmaceutical industries throughout the past decades. Despite their typically low growth rate, unstable productivity and high process costs, mammalian cells benefit from their ability to perform human-like posttranslational modifications and thus represent the main expression system for mAb production [1–3]. Chinese hamster ovary (CHO) cells are the most commonly used cell systems for mammalian processes. Since GMP certificated processes are readily available and high throughput cell screening and fed-batch processing are established and well understood CHO cells will remain the main expression system in the near future [3–5]. Advanced understanding of CHO cell metabolism and interaction with process parameters is of high importance for process optimization, scale-up; and moreover, is a major requirement of quality by design (QbD) guidelines as claimed by the Federal Drug Association (FDA) [6]. Furthermore, modeling approaches as hybrid models may benefit from the knowledge of process parameter interactions and single effects to improve model fit and model quality [7]. Understanding of process parameter interactions is especially useful during process scale-up, where unwanted variations of pH, dissolved oxygen tension (*p*O_2_) and carbon dioxide tension (*p*CO_2_) are most likely to occur [8–10]. Manufacturing processes aim towards high product titers and space–time-yields but, moreover, good and consistent final product quality is of upmost importance. Monoclonal antibodies are very complex molecules and can be modified during up- and downstream process thus leading to heterogeneous final products [11]. Product heterogeneity may lead to various changes in physiochemical, biological and immunogenic properties compared to the desired homogeneous antibody drug. In more detail, protein heterogeneity may lead to different protein binding, stability, immune responses and pharmacokinetics [12]. Therefore, control of product heterogeneity within predefined analytical specifications is of high importance for cGMP manufacturing.

Process parameters such as temperature, pH, *p*O_2_ and *p*CO_2_ can have enormous effects on CHO cell process performance and critical quality attributes as described widely in the literature [13–17]. Link et al. [15] discovered that changing dissolved oxygen concentration can have an effect on specific productivity of CHO-K1 cells. Additionally, a study conducted by Trummer et al. [13] showed significant influences of culture temperature and culture pH on cell metabolism, growth and product quality in a CHO batch fermentation producing EPO-Fc. However, influences of different carbon dioxide tensions are most often neglected although it has been shown in other studies that *p*CO_2_ can have significant effects on specific cell growth, productivity and product quality [18–20]. Studies about interaction effects of parameters are less prominent in the literature, however, Zanghi et al. [19] showed that at elevated pH or *p*CO_2_ in a CHO culture, osmolality and bicarbonate concentration significantly influence product polysialylation.

In contrast to the current state of the art, the goal of our study was to derive interaction effects of critical scale-up parameters on cell physiology as well as on process performance and critical product quality attributes. Therefore, our approach consisted of a central composite face-centered design of experiments and various in-process analytics as well as product quality analytics for charge, size and glycan heterogeneity. This investigation was only possible through usage of a novel control strategy via decoupling of *p*O_2_, pH and *p*CO_2_ process control and thus investigating the individual effects and interactions of these critical scale-up parameters in one systematic approach. The novelty of this work is therefore the elucidation of interaction effects of scale dependent process parameters on a large number of responses including amino acids and product quality attributes.

## Materials and methods

### Design of experiment and data evaluation

Experiments were carried out according to a central composite face-centered design with the factors pH ranging from 6.8 to 7.2, *p*O_2_ from 10 to 40 % and *p*CO_2_ from 5 (37 mmHg) to 20 % (150 mmHg) (Table 1). Boundaries were set for *p*CO_2_ in between physiological values and levels that might occur during large-scale fermentation processes [9], whereas pH was mostly set in between boundaries of physiological ranges to allow constant fermentation conditions. However, pH variations to 6.8 may also occur during large-scale fermentation due to CO_2_ gradients [21]. The dissolved oxygen tension concentration upper limit was set to 40 % according to common industrial settings. Since in large-scale fermentations at high cell densities *p*O_2_ gradients are likely to occur [9], the lower limit for *p*O_2_ was set to 10 %. In total, the experimental design space consisted of 19 batch fermentations, whereas five fermentation runs were carried out at the center point settings of the DoE. Due to problems with one of the pH-probes, one fermentation run at pH 7.2, *p*O_2_ 40 % and *p*CO_2_ 20 % finally had to be excluded from data evaluation. Since all other 14 set points of the experimental design space were carried out successfully, this one missing run was found not to compromise the quality of the experimental design to any significant extent. Furthermore, two fermentation runs (pH 7; *p*CO_2_ 20 %; *p*O_2_ 25 % and pH 7; *p*CO_2_ 12.5 %; *p*O_2_ 10 %) revealed unusually high mAb aggregation rates and thus their mAb quality data were excluded from data evaluation.

**Table 1:** Specific process parameter conditions of the experimental design space

Exp. No.	1	2	3	4	5	6	7	8	9	10
pH	6.8	7.2	6.8	7.2	6.8	7.2	6.8	7.2	7	7
*p*CO_2_	5	5	20	20	5	5	20	20	12.5	12.5
*p*O_2_	10	10	10	10	40	40	40	40	25	25

Exp. No.	11	12	13	14	15	16	17	18	19	
pH	7	7.2	6.8	7	7	7	7	7	7	
*p*CO_2_	12.5	12.5	12.5	5	20	12.5	12.5	12.5	12.5	
*p*O_2_	25	25	25	25	25	10	40	25	25	

Experiment number 8 had to be excluded from data evaluation due to a defect pH probe

Experimental planning and data evaluation was carried out by the help of the software MODDE^®^ (Umetrics, Sweden). For each selected response MODDE^®^ generates a PLS Model and the final parameters after model optimization are illustrated in this report. Key parameters of the models are the coefficient of determination *R* ^2^, goodness of prediction *Q* ^2^, model validity MV and reproducibility RP. Ideally, *R* ^2^, *Q* ^2^, MV and RP should be close to 1 and above 0.5, or 0.25 concerning MV, for a significant model. Furthermore, tables with normalized coefficients derived out of PLS models are shown in this work. Since errors for specific growth and metabolite production/consumption rates are generally high in mammalian processes [22], confidence intervals were set to 0.90 for all calculated growth, consumption and production rates. For all other responses, confidence levels were set to 0.95. The responses analyzed were maximum and average specific growth rate (*µ*_max_, *µ*_average_), specific lactate and ammonia production (*q*_lac_, *q*_amm_), specific glucose and glutamine consumption (*q*_gluc_, *q*_gln_), further specific amino acid consumption and production rates (*q*_Ala_, *q*_Arg_, *q*_Asn_, *q*_Asp_, *q*_Cys_, *q*_Glu_, *q*_Gly_, *q*_His_, *q*_Ile_, *q*_Leu_, *q*_Lys_, *q*_Meth_, *q*_Phe_, *q*_Pro_, *q*_Ser_, *q*_Thr_, *q*_Tyr_, *q*_Try_, *q*_Val_) and specific productivity (*q*_p_). Moreover, critical product quality attributes were analyzed as charge, size and glycan variants.

### Cell line, seed train and batch fermentations

An industrial CHO cell line producing a mAb was cultivated in chemically defined media. Precultures for batch fermentation processes were cultivated in shake flasks and incubated at 10 % *p*CO_2_ and 37 °C temperature. Exponentially growing cells were transferred into 3 L glass bioreactors resulting in an inoculation density of 3 × 10^5^ cells/mL. All batch cultivations were carried out at 37 °C.

Using a novel control strategy, *p*O_2_, *p*CO_2_ and pH set points were set and kept constant throughout the fermentation process according to the DoE. Usually, pH control in cell culture is performed using *p*CO_2_ as acid; this had to be changed to decouple pH and *p*CO_2_ control. pH regulation was therefore realized by the addition of 0.5 M HCL and 0.5 M NaOH, respectively. Dissolved oxygen tension and carbon dioxide tension were regulated independently through gas mixing while keeping stirrer speed and gas volumetric flow rate constant. *p*CO_2_ measurement and control was done by use of an off-gas sensor (BlueInOne, Bluesens, Germany) and based on calculations from Frahm et al. [23]. pH and *p*O_2_ were measured by in-line probes (EasyFerm, Hamilton, United States and VisiFerm, Hamilton, United States).

### In-process control, mAb and amino acid determination

Cultivation samples were taken every 12 h and cell counting/viability determination was performed using the automatic picture analyzer Cedex HiRes Analyzer (Roche, Germany). Osmolality of supernatant was determined via freeze point depression (Mikro-Osmometer TypOM806, Löser, Germany). Analysis of metabolites glucose, glutamine, glutamate, lactate and ammonium were performed using Cedex Bio HT Analyzer (Roche, Germany). Antibody titer determination was carried out by HPLC (Ultimate 3000, Dionex, United States) with a Protein A sensor cartridge (Applied Biosystems, The Netherlands). Amino acid concentrations were determined by HPLC measurement (Ultimate 3000, Dionex, United States; ZORBAX Eclipse Plus C18 column, Agilent Technologies, United States) and prior sample-derivatization with ortho-phthalaldehyde (OPA) and 9-fluorenylmethyloxycarbonyl (FMOC).

### Product quality analytics

Harvest samples for product quality analytics were taken only once at the end of batch processes as soon as viability dropped below 75 % and supernatants were stored at −80 °C.

#### Cation exchange chromatography

Determination of charge variants was performed using a ProPac WCX-10 (4 × 250 mm) analytical column (Dionex, United States) connected to HPLC system (Agilent Technologies 1100/1200 Series, United States) with UV detection at 220 nm.

#### Size exclusion chromatography

Size exclusion chromatography was performed using a TSKgel G3000SWXL column (Tosoh, Japan) connected to a HPLC system (Agilent Technologies 1100/1200 Series, United States) with UV detection at 210 nm.

#### *N*-glycan determination

Quantitative determination of *N*-glycans was performed after digest with *N*-glycosidase F (PNGase F, Roche, Germany). After separation from the protein using ultra centrifugal filters, released *N*-glycans were labeled with 2-aminobenzamide (2-AB) at 37 °C overnight. Afterwards 2-AB labeled glycans were profiled by normal phase chromatography using a ACQUITY UPLC BEH column (Waters, United States) connected to an HPLC system (Agilent 1200 series, United States) with FLD detection.

### Calculation of specific rates and degree of glycosylation

Calculation of the specific growth rate µ was performed using the integral of viable cell density as described by Klein et al. [24] and shown in Eq. (1):

1 $$\mu = \frac{\text{d(VCD)}}{\text{d(IVCD)}},$$

with VCD being the viable cell density and IVCD the integral of viable cell density.

Calculation of the specific production or consumption rates was performed as shown in Eq. (2), with c_i_ being the concentration of either a specific metabolite or product *i*:

2 $$q_{i} = \frac{{{\text{d}}(c_{i} )}}{{{\text{d(IVCD}})}}.$$

Calculation of the specific growth and production or consumption rates were performed for each time point and average rates were calculated for the relevant process phase using values between inoculation and peak VCD. The maximum specific growth rate, however, was calculated only for time points within the exponential growth phase of the processes. All cell specific metabolic rates were assigned positive for production and negative for consumption.

The degree of galactosylation (GI) was calculated as shown in Eq. (3) and described earlier by Ivarsson et al. [17]:

3 $${\text{GI}} = \frac{3 \times G3 + 2 \times G2 + G1}{(G0 + G1 + G2 + G3) \times 3}.$$

The degrees of sialylation (SI) and afucosylation (aFI) were calculated in the same manner.

## Results and discussion

Experiments were carried out according to our experimental design as specified in the “Materials and methods” section. The specific data points for the PLS models are summarized in the supplementary file. Concerning the evaluation of the data, additionally to our controlled process parameters, the influences of important uncontrolled parameters on the responses of the design of experiments were considered. Especially effects that might occur due to different osmolalities, average cell viabilities and overall process time.

Although only minor variations in osmolality occurred during cell growth (mostly between 290 and 330 mOsm/kg), the influence of variations in osmolality was investigated in separate experiments and no significant differences in cell specific growth, productivity or metabolite production/consumption could be derived for this cell line (data not shown).

**Fig. 1:**
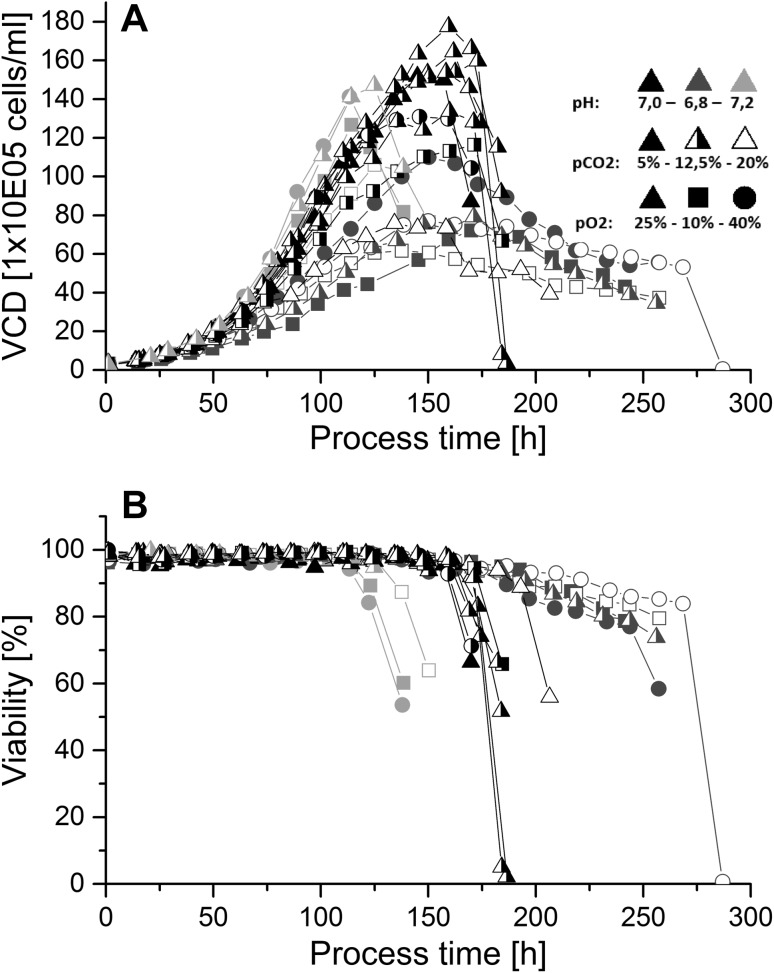
Viable cell density (**a**) and cell viability (**b**) over process time for all batch fermentations. (*Black symbols* represent processes at pH 7.0, *blue symbols* at pH 6.8, *red symbols* at pH 7.2; *closed symbols* represent processes at *p*CO_2_ 5 %, *half-closed* at 12.5 % and *open symbols* at 20 %; *triangles* represent processes at *p*O_2_ 25 %, *squares* at 10 %, *circles* at 40 %). High pH values led to high viable cell densities but concurrently to shorter process time due to faster depletion of the main c-source. Cell viabilities stayed at high values as long as glutamine was available

The overall batch process time and mean cell viabilities in this study correlate strongly with process pH but not with *p*O_2_ and *p*CO_2_ (Fig. 1). Therefore, effects on product quality data attributed to process pH might furthermore derive from different process times or mean cell viabilities. Effects of *p*CO_2_ and *p*O_2_ were found to be independent from process time. The authors would like to state that due to the complexity of mammalian cell culture processes, only a certain portion of the data variability can be explained by the presented models. Therefore, the goodness of fit *R* ^2^, a measure how good the model fits the observed data, and the goodness of prediction *Q* ^2^, an estimate of the predictive ability of the model, deviate from the ideal value of 1. However, *R* ^2^ and *Q* ^2^ values are presented in the corresponding data tables and only significant models with values above 0.5 are discussed.

### Effects on cell growth and viability

Cell growth and cell viability are essential parameters that have to be monitored closely during mammalian fermentation processes. This is usually done by automated offline cell counting and live/dead cell staining. Through determination of viable and total cell densities (VCD and TCD) specific cell growth and cell viabilities were evaluated.

**Fig. 2:**
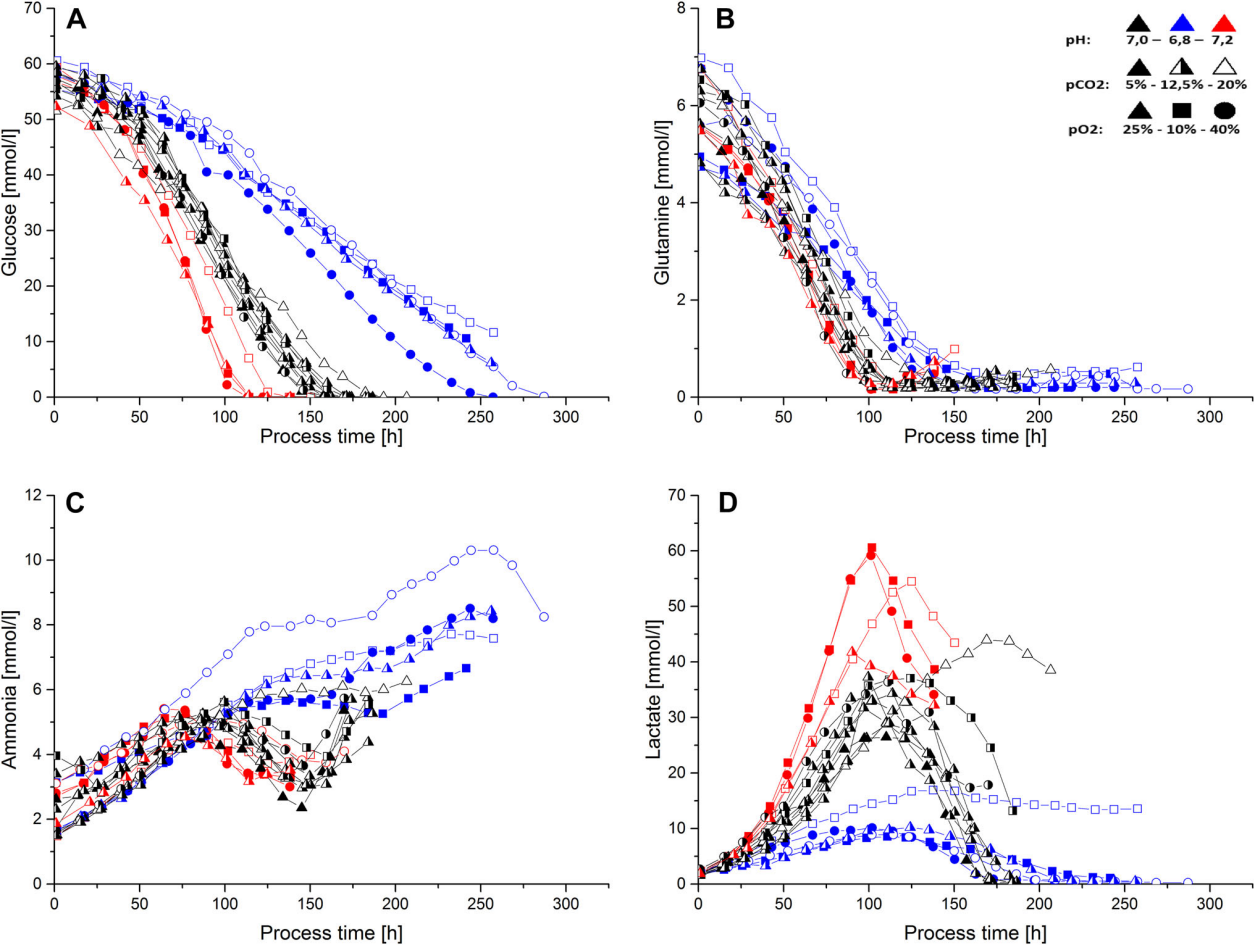
Metabolite profiles for all conducted batch processes. (*Black symbols* represent processes at pH 7.0, *blue symbols* at pH 6.8, *red symbols* at pH 7.2; *closed symbols* represent processes at *p*CO_2_ 5 %, *half-closed* at 12.5 % and *open symbols* at 20 %; *triangles* represent processes at *p*O_2_ 25 %, *squares* at 10 %, *circles* at 40 %). **a** Glucose became limiting in almost all fermentations before reaching the harvest criteria of 75 % viability. **b** Glutamine profiles showed glutamine limitations after 100–150 h of process time for all batches. **c** Ammonia concentrations showed process phases of production and consumption for almost all runs at pH values of 6.8 and 7.0, whereas only production and steady-state values were derived from fermentation runs at pH 6.8. **d** Lactate was produced and consumed during all processes

Comparing the maximum viable cell density (VCD) of all batch processes, it can be derived that processes at pH 7.0 reached the highest maximum viable cell densities (Fig. 1a). All processes declined in viabilities to values lower than 75 % shortly after limitation of the main C-source. Therefore, differences in maximum VCD derived not only from different growth behavior but furthermore from nutrient availability. Cell viabilities stayed at high values and decreased dramatically as soon as glucose became limiting. Furthermore, slight decreases in cell viabilities could be detected for processes at pH 7.0 and 6.8, most probably due to glutamine limitation before final glucose depletion (Figs. 1b, 2a/b).

Regarding specific cell growth during exponential growth (*µ*_max_) and total growth phase (*µ*_average_) significant influences of pH, *p*O_2_ and *p*CO_2_ as well as a significant interaction of pH and *p*CO_2_ could be detected (Table 2). *µ*_max_ and *µ*_average_ were strongly affected by process conditions, whereby the lowest growth rates were reduced to around 35 and 45 %, respectively, when compared to the maximum values obtained in this DoE.

**Table 2:** Coefficient table and summary of fit of the models obtained for specific growth rates, specific glucose consumption, specific lactate and IgG production rates

	pH	*p*O_2_	*p*CO_2_	pH^2^	*p*O_2_^2^	*p*CO_2_^2^	pH × *p*CO_2_	*R* ^2^	*Q* ^2^	MV	RP
*µ*_average_	0.83	0.28	−0.18	–	–	–	−0.18	0.88	0.70	0.93	0.79
*µ*_max_	0.67	0.32	−0.18	−0.19	−0.21	−0.22	−0.23	0.95	0.82	0.87	0.90
*q*_glucose_	−0.85	–	–	−0.26	–	–	–	0.82	0.78	0.65	0.89
*q*_lactate_	0.93	–	–	–	–	–	–	0.87	0.85	0.98	0.65
*q*_p_	0.58	0.34	−*0.14*	–	–	−0.31	−0.28	0.76	0.55	0.63	0.81

The coefficients according to the individual factors of the DoE are normalized. Coefficients that are shown in italic are not considered as significant regarding the applied significance level

Table 2 shows that pH affected specific cell growth the most and higher pH values led to higher cell growth. Increased *p*CO_2_ had a negative impact on cell growth, whereas increased *p*O_2_ seemed to stimulate specific cell growth between the borders of our experimental design. These general results confirm other publications as Link et al. [15], Yoon et al. [25], Trummer et al. [13] and Dezengotita et al. [18]. Remarkably, through the independent control of process parameters our study derived additional significant interaction effects of pH and *p*CO_2_ on specific growth.

### Effects on cell metabolism and productivity

CHO cell metabolism strongly depends on the main carbon and energy sources, glucose and glutamine. Furthermore, by-product accumulation can be of high interest since lactate and ammonia concentrations can affect cell physiology at elevated levels [26]. Moreover, specific productivity of cells is clearly of high importance, because final product titer levels are directly correlated with this entity. Additionally, amino acids are one of the most important cell culture media components, influencing cell growth and productivity [27].

In Table 2 the result for the DoE evaluation regarding the average specific glucose uptake rate *q*_gluc_ is shown. *q*_gluc_ was significantly influenced by process pH whereby lowest rates were reduced to around 60 % when compared to the maximum consumption rates obtained in this DoE. No significant *p*O_2_, *p*CO_2_ or quadratic/interaction terms could be determined. These results can be confirmed by other studies [13, 25, 26].

The mean specific glutamine uptake rate was not significantly influenced by pH or *p*CO_2_ or *p*O_2_ (data not shown). Concerning pH this is in accordance to the results from Yoon et al. [25] but contrary to Trummer et al. [13] who observed higher specific consumption rates at higher pH values. Processes at high pH values depleted earlier of glutamine than processes at low pH (Fig. 2). Since no effects on specific glutamine uptake rates could be determined, this effect is most probably due to higher viable cell densities and thus higher total glutamine consumptions at high pH values.

The average specific lactate production rate *q*_lac_ was significantly affected by process conditions (Table 1), whereby lowest rates were reduced to around 80 % when compared to the maximum production rates obtained in this study. Comparing the specific lactate production *q*_lac_ during the growth phase, data clearly shows a direct link between pH and lactate production. Runs at highest pH values produced significantly more lactate than cells at lower pH. This effect is well reported in literature [13]. Furthermore, the yield of glucose to lactate was affected by culture pH, higher pH values leading to higher yields. Dependencies of *q*_lac_ from *p*O_2_ or *p*CO_2_ could not be found.

No significant effects of either pH, *p*CO_2_ or *p*O_2_ on specific ammonia production could be observed (data not shown). This is in agreement with the results from Yoon et al. [25] but contrary to Trummer et al. [13], who found a dependency of specific ammonia production from culture pH. The contrary findings on specific ammonia production and glutamine consumption in the stated literature may indicate that effects on ammonia and glutamine metabolism are more cell line specific than effects on glucose and lactate metabolism.

Due to higher specific glucose consumption rates and higher cell growth, processes became glucose-limited earliest at pH 7.2 (Fig. 2a). Ammonia profiles varied between phases of ammonia production and consumption throughout most processes at 7.0 (Fig. 2c). All fermentation runs at pH 6.8 showed no significant ammonia consumption over process time and thus resulted in general higher final ammonia concentrations. Similar to Li et al. [28] low levels of lactate during the lactate consumption phase, present at most processes at pH 7.0 and 6.8, led to the consumption of alanine (data not shown) and subsequently to an increase in final ammonia levels.

Additionally to glutamine, 19 other amino acids were analyzed by HPLC. Specific consumption or production rates were calculated during the growth phase and significant models could be derived for nine amino acids (Table 3). Hereby, higher pH values led to a higher consumption of several amino acids (Ser, Asp, Val, Ile, Arg and His). Similar effects were reported from Trummer et al. [13]. Furthermore, significant effects of *p*O_2_ and *p*CO_2_ on various amino acid consumption rates could be derived indicating higher consumption rates at process conditions that were favorable for cell growth (*µ*_max_, *µ*_average_). To our knowledge, no effects of *p*O_2_ or *p*CO_2_ on specific amino acid consumption or production rates in CHO cells have been shown before in the literature. Seven out of nine specific amino acid consumption or production rates were significantly affected by process parameter interactions; this demonstrates that interaction effects of process parameters on cell physiology have to be considered.

**Table 3:** Coefficient table and summary of fit of the models obtained for specific amino acid production and consumption rates

	pH		*p*O_2_	*p*CO_2_	pH^2^	*p*CO_2_^2^	pH × *p*CO_2_	pH × *p*O_2_	*R* ^2^	*Q* ^2^	MV	RP
*q*_Asp_	−0.58		−0.2	−0.25	–	0.67	–	–	0.96	0.82	0.91	0.91
*q*_Glu_	–		–	−0.76	–	0.39	–	–	0.76	0.69	0.90	0.66
*q*_Ser_	−0.45		–	−0.47	−*0.31*	−*0.22*	0.26	–	0.82	0.58	0.90	0.67
*q*_His_	−0.36		−0.37	−*0.13*	−*0.29*	–	0.37	–	0.75	0.52	0.75	0.71
*q*_Arg_	−0.67		−0.59	−*0.2*	–	−0.2	–	0.24	0.93	0.72	0.79	0.90
*q*_Gly_	−*0.20*		−0.58	0.37	–	–	–	0.42	0.81	0.63	0.52	0.89
*q*_Cys_	−0.31		−0.57	–	–	–	–	0.49	0.78	0.70	0.77	0.78
*q*_Val_	−0.58		–	*0.12*	–	−0.49	0.32	–	0.94	0.61	0.57	0.95
*q*_Ile_	−0.44		–	*0.20*	–	−0.31	0.47	–	0.77	0.51	0.33	0.92

*q*_Gly_ and *q*_Cys_ refer to specific amino acid production and all other amino acids to consumption rates. The coefficients according to the individual factors of the DoE are normalized. Coefficients that are shown in italic are not considered as significant regarding the applied significance level

Amino acids could be divided into three groups. The first group consisted of amino acids that were only consumed throughout the batch processes (Arg, Val, Phe, Iso, Leu, Pro, Asn, Ser, His, Tyr, Lys, Trp and Met), the second contains amino acids that were only produced over process time (Gly, Cys, Glu) and the last one consists of aspartate and alanine, which were produced and/or consumed during the batch processes (data not shown). In contrast to Trummer et al. [13], aspartate was only produced at lower pH values. Processes were not limited in any amino acid before process harvest except of glutamine for processes at pH 7.0 and 6.8 (Fig. 2b). Similar to effects reported from Zagari et al. [29] and Wahrheit et al. [30] cells reacted to glutamine limitation via uptake of alternative carbon sources as lactate and aspartate, except processes at pH 7.0, *p*CO_2_ 20 %, *p*O_2_ 25 % and pH 6.8, *p*CO_2_ 20 % and *p*O_2_ 10 % which showed no lactate consumption or very low consumption after glutamine limitation (Fig. 2d).

The average specific IgG production rate *q*_P_ was significantly affected by process conditions, whereby lowest rates were reduced to around 30 % when compared to the maximum production rates obtained in this study.

The model data out of Table 2 indicates that pH strongly affected specific productivity in a way that high pH set points led to high *q*_p_ values. Furthermore, quadratic effects of *p*CO_2_, linear effects of *p*O_2_ and an interaction term of *p*CO_2_ and pH were identified. Positive effects of *p*O_2_ on *q*_*p*_ are also reported in Link et al. [15], whereas Trummer et al. [13] found no connections between *p*O_2_ and *q*_p_. Concerning *p*CO_2_ Gray et al. [20] showed optimum levels around 76 mmHg, which is in agreement with our findings. No effects of culture pH on *q*_p_ could be shown in studies from Trummer et al. [13] and Yoon et al. [25]. However, other authors reported similar results, whereby *q*_p_ increased with increasing pH between pH 6.8 and 7.2 [31]. Contradictions between the stated findings might derive from the different cell lines used in these studies. Additionally, pH and *p*CO_2_ interaction effects similar to those already observed for *µ*_average_ and *µ*_max_ could be detected for *q*_p_. To our knowledge, no interaction effects of pH and *p*CO_2_ on cell specific productivity have been reported so far in the literature. The derived model showed similar coefficients to that one obtained for *µ*_max_ and *µ*_average_. This indicates that fermentation conditions that induce high specific growth rates also induce high specific production rates for this cell line.

**Fig. 3:**
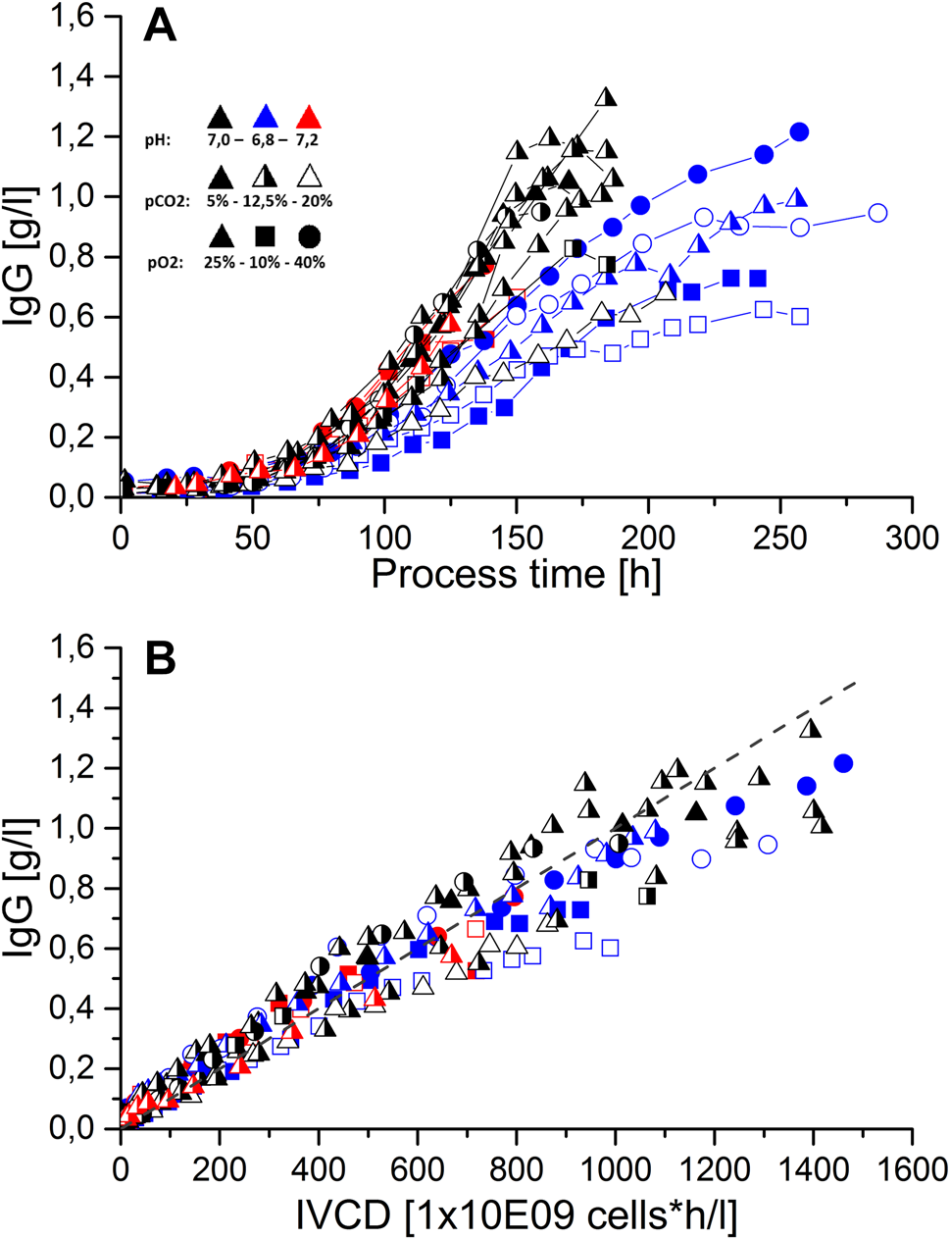
IgG concentration over process time (**a**) and integral viable cell density (**b**) for all batch fermentations. (*Black symbols* represent processes at pH 7.0, *blue symbols* at pH 6.8, *red symbols* at pH 7.2; *closed symbols* represent processes at *p*CO_2_ 5 %, *half-closed* at 12.5 % and *open symbols* at 20 %; *triangles* represent processes at *p*O_2_ 25 %, *squares* at 10 %, *circles* at 40 %). Highest process titers were obtained for fermentation runs conducted at pH 7.0 and 6.8, mainly due to the highest IVCD values at these process conditions

Overall process titer of batch processes was mostly dependent from the integral viable cell density similar to Trummer et al. [13]. Therefore, highest final product concentrations could be derived at pH 7.0 and 6.8, whereas space–time-yields were higher for processes at pH 7.0 (Fig. 3).

### Effects on critical quality attributes (CQAs)

#### Size exclusion chromatography (SEC) for determination of antibody size heterogeneity

During manufacturing and storage antibody size variants (e.g., aggregates and fragments) occur. Since size variants can influence immunogenicity, potency and pharmacokinetics their levels are monitored during lot release, stability and characterization [32].

Data out of SEC analysis show minor variations with overall purity levels between 96 and 98 % relative Area (data not shown). PLS models for sum of aggregates and sum of fragments were conducted. No dependencies of *p*CO_2_, *p*O_2_ or pH on antibody aggregation or fragmentation were obtained from the gathered model data. Jing et al. [33] observed a significant increase in protein aggregation when changing dissolved oxygen from 50 to 15 % air saturation. Concerning pH, values far away from the isoelectric point of the desired antibody showed better protein solubility [34, 35, 36]. In contrast to Gomes and Hiller [37], we could not detect any changes in protein aggregation when varying culture conditions in between the DoE settings. The mechanisms leading to product aggregation may be strongly product specific, explaining the differences between the results in the literature.

#### Cation exchange chromatography (CEX) for determination of antibody charge heterogeneity

Charge based antibody characterization is a frequently used tool since it is sensitive to many type of modifications as protein conformation, size, sequence species, glycosylation and posttranslational modifications [11]. Therefore, various antibody variations that occur with changing process conditions were detected.

**Fig. 4:**
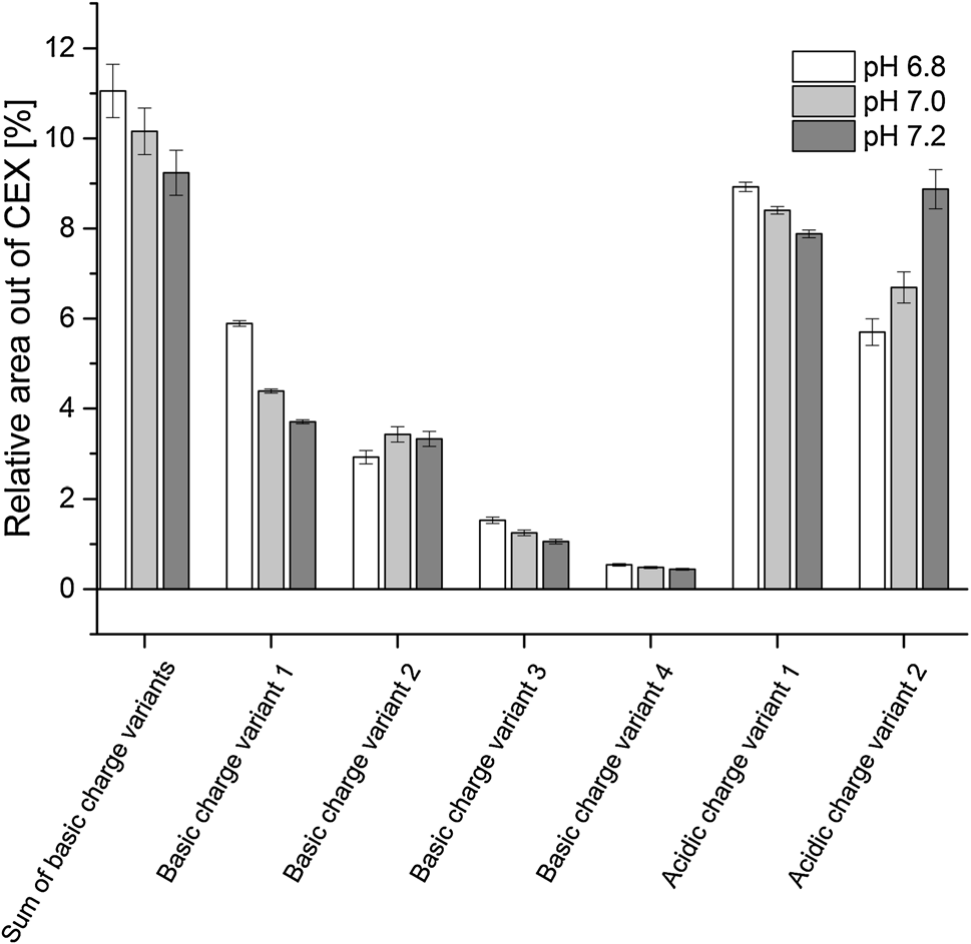
Relative area out of cation exchange chromatography (CEX) for various charge variants and one sum parameter at different pH set points for *p*CO_2_ 12.5 % and *p*O_2_ 25 %

In total, we detected 18 different charge variants, whereby significant models could be obtained for six individual variants (i)–(vi) and one sum parameter (Fig. 4; Table 4).

**Table 4:** Coefficient table and summary of fit of the models obtained for the mAb charge variants and one glycan variant

	pH	*p*O_2_	*p*CO_2_	pH^2^	*p*O_2_^2^	*p*CO_2_^2^	pH × *p*CO_2_	*R* ^2^	*Q* ^2^	MV	RP
(i) ACV 1	−0.44	–	*0.37*	*0.24*	–	*0.28*	−*0.22*	0.67	0.57	0.78	0.67
(ii) ACV 2	0.94	–	−*0.04*	–	–	–	−0.15	0.92	0.84	0.94	0.86
(iii) BCV 1	−0.65	*0.13*	*0.17*	–	*0.27*	*0.29*	–	0.87	0.72	0.59	0.90
(iv) BCV 2	0.74	–	–	−0.58	–	–	–	0.72	0.60	0.38	0.90
(v) BCV 3	−0.88	–	–	–	–	–	–	0.77	0.73	0.68	0.85
(vi) BCV 4	−0.73	–	0.41	–	–	–	–	0.78	0.71	0.78	0.81
sum of basic variants	−0.64	*0.19*	*0.17*	–	*0.22*	*0.3*	–	0.82	0.59	0.64	0.84
bG1FSA_2	−*0.03*	–	−*0.26*	0.67	–	–	−0.48	0.76	0.53	0.84	0.68

The coefficients according to the individual factors of the DoE are normalized. Coefficients that are shown in italic are not considered as significant regarding the applied significance. Acidic charge variants (ACV), basic charge variants (BCV) and one glycan variant are presented

(i) The content of acidic charge variant 1 (deamidation of asparagine to aspartate on one light chain) was significantly influenced by culture pH (Table 4). Deamidation is an unavoidable alteration reaction that takes place in fermentation broth after secretion of the cell. Deamidation can finally contribute to heterogeneity; affect protein crystallization, stability and efficacy [38, 39]. Therefore, Liu et al. [40] considered Asn deamidation as one of the most important common modifications for mAbs. Deamidation in our batch process data differed significantly, whereas at pH 6.8 the highest protein deamidation could be observed (Fig. 4). In contradiction to the literature, our studies would suggest that lower pH values lead to higher deamidation rates [41].

(ii) Considering acidic species 2 lower pH values led to lower acidic variants (Fig. 4; Table 4). Additionally, a significant interaction term for pH and *p*CO_2_ affecting acidic variant 2 was identified.

(iii) Isomerization of Asp on one heavy chain, basic charge variant 1, is congeneric to the deamidation reaction leading to acidic charge variant 1. The isomerization reaction occurs spontaneously in the culture media [38]. Data shows that pH had a significant effect on isomerization whereby at lower pH values, higher amounts of isomerized variants occurred (Fig. 4; Table 4). These findings are in agreement with the literature [42]. Analogous to Asn deamidation, Asp isomerization is considered as one of the most important common modifications for mAbs [40].

(iv, v) The presence of lysine residues on one heavy chain, basic charge variant 2, was significantly affected by culture pH (Fig. 4; Table 4). Moreover, the amount of lysine residues on both heavy chains, basic charge variant 3, was also significantly affected by culture pH but with opposite outcome (Fig. 4; Table 4). For charge variant 2, lower culture pH led to lower amount of lysine residues compared to runs at higher pH values. In contrast, models obtained for charge variant 3 showed the opposite effect. C-terminal lysine residues are a very common modification observed during monoclonal antibody production. After cell lysis, the release of basic carboxypeptidase is supposed to be the reason for lysine heterogeneity, since no spontaneous reactions were found in other studies [39, 43–45]. Lower cell viabilities and prolonged process time at pH 6.8, therefore, should have led to a better cleavage of lysine residues from the antibody. Interestingly, this is only the case for lysine residue 2 whereas for charge variant 3 the opposite effect is visible. C-terminal lysine residues are considered as a rather less important mAb modification [40].

(vi) The amount of basic charge variant 4 was significantly influenced by process pH. The higher the pH value, the lower the amount of basic charge variant 4 (Fig. 4; Table 4). Moreover, a significant linear correlation with *p*CO_2_ could be conducted out of the PLS model.

#### Glycosylation profile analysis for determination of *N*-glycosylation heterogeneity

Proper glycosylation of mAbs is of upmost importance since it can influence stability, effector functions, immunogenicity and pharmacokinetics of the desired product [17]. 21 different glycosylation patterns could be identified whereby the only significant model concerning data from glycosylation analysis could be obtained for glycosylation pattern bG1FSA_2 (di-*N*-acetylneuraminic acidylated, mono-galactosylated, biantennary, fucosylated) (Table 4). Significant single effects of process pH and an interaction term with *p*CO_2_ on glycosylation variant bG1SA_2 were obtained. Analogous to Zanghi et al. [19], these results would suggest a lower sialylation at higher dissolved carbon dioxide and higher pH values. Around 75 % of the observed glycosylation profiles consisted of bG0F (biantennary, fucosylated) and bG1F (biantennary, mono-galactosylated, fucosylated) independent from process conditions similar to Agarabi et al. [46] and as reported by Raju et al. [47]. Furthermore, galactosylation, sialylation and afucosylation level (GI, SI, aFI) variations stayed in between narrow limits, mostly 25–30 % GI, 0.5–1.5 % SI and 4–8.0 % aFI, for all batch processes. Moreover, GI, aFI and SI variation between center point runs covered most glycosylation variations observed.

**Fig. 5:**
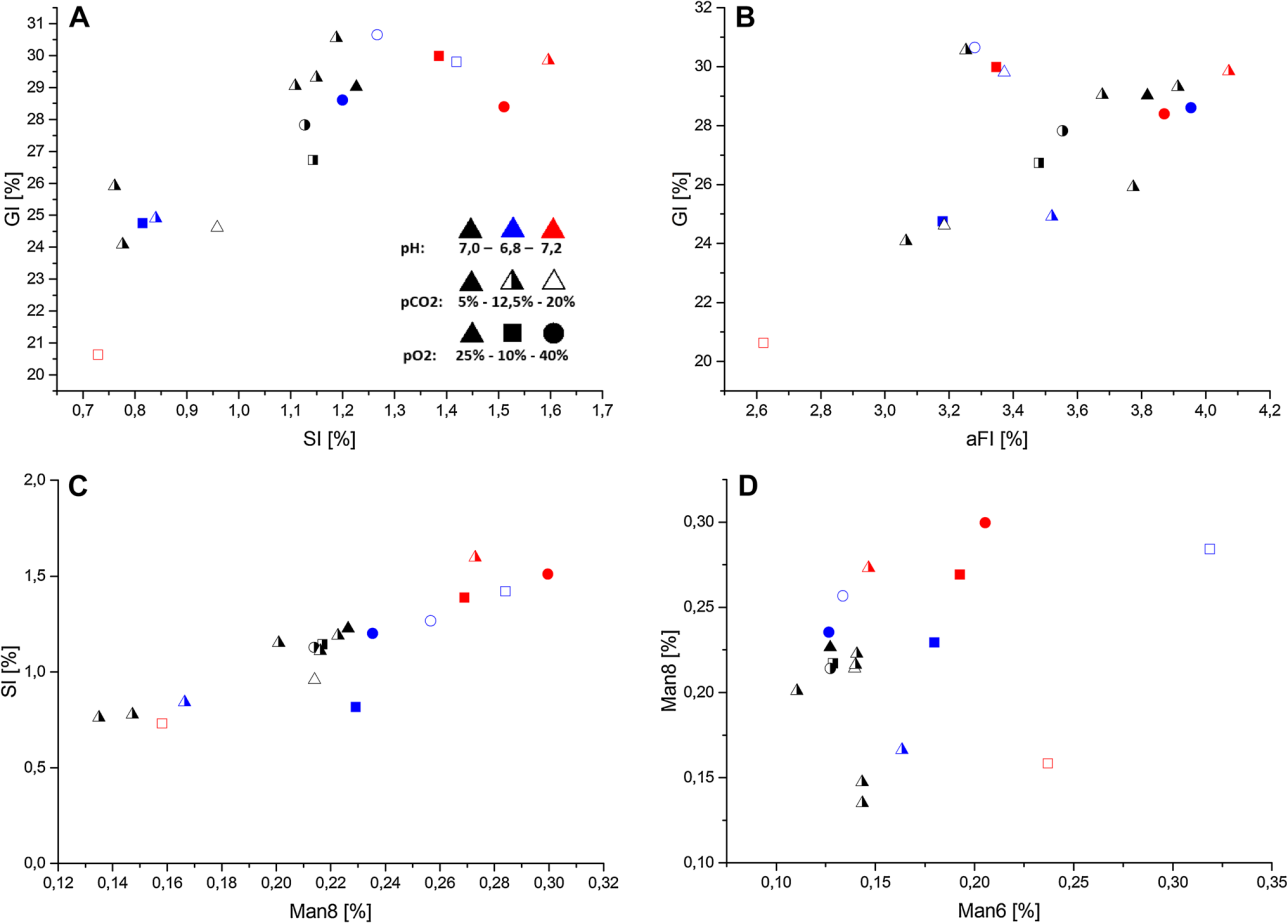
Correlations and trends of glycosylation variants. (*Black symbols* represent processes at pH 7.0, *blue symbols* at pH 6.8, *red symbols* at pH 7.2; *closed symbols* represent processes at *p*CO_2_ 5 %, *half-closed* at 12.5 % and *open symbols* at 20 %; *triangles* represent processes at *p*O_2_ 25 %, *squares* at 10 %, *circles* at 40 %). Antibody galactosylation level (GI) over **a** sialylation (SI) and **b** afucosylation (aFI). Antibody galactosylation seemed to correlate positively with afucosylation and sialylation. A strong correlation between sialylation and mannosylation 8 were derived from C. In D, mannose 8 levels are plotted over mannose 6, it can be derived that highest mannosylation levels only occurred for processes at pH 6.8 and 7.2

When plotting GI over SI and aFI, GI values correlate positively with aFI and SI values (Fig. 5a linear determination coefficient *R* ^2^ = 0.66; Fig. 5b linear determination coefficient *R* ^2^ = 0.36). This indicates that afucosylation, sialylation and galactosylation were all influenced likewise by process conditions. Additionally, highest SI levels could be observed mostly for pH values of 7.2. Furthermore, lowest SI levels did not occur for high *p*O_2_ values at 40 %. Regarding sialylation and process pH similar trends could be detected from Ivarsson et al. [17]. Moreover, high sialylation correlated with high Mannose 8 (Fig. 5c linear determination coefficient *R* ^2^ = 0.82) variants but not with Mannose 6 variants. Finally, highest amounts of mannose 6 and 8 variants could only be observed for pH values at 6.8 and 7.2 (Fig. 5d). A huge amount of process variables affecting protein glycosylation in mammalian cells have been reported as substrate concentrations, media composition, by-product accumulation, temperature, cell viability and shear stress [48]. Literature about the influence of process parameters on mAb glycosylation are partly contradicting and seem to be strongly dependent on the specific cell line, product and cultivation conditions. Concerning *p*O_2_ variations different results are reported but consistent glycosylation profiles can be expected for DO between 10 and 100 % [48]. Ivarsson et al. [17] stated a slight increased protein galactosylation and sialylation at low DO 10 % and high DO 90 % compared to 50 % DO. Studies about the influence of *p*CO_2_ on glycosylation are less represented. Nevertheless, Zanghi et al. [19] and Kimura and Miller [49] showed decreased polysialylation and *N*-glycolylneuraminic acid (NGNA) when increasing *p*CO_2_. Trummer et al. [13] reported no effect on EPO-FC sialylation when varying DO between 10 and 100 % and pH between 6.8 and 7.3. In contrast, Ivarsson et al. [17] recently showed that galactosylation and sialylation levels slightly increased when pH increased between 6.8 and 7.2.

Finally, through our applied control strategy and experimental design, we could not only detect independent single process parameter effects on cell physiology and product quality but also furthermore derive several new process parameters interaction effects. A short summary of several key responses affected by process parameter interactions is given in Table 5.

**Table 5:** Summary table of key responses affected by process parameter interactions as well as the observed single parameter effects and literature comparison

	Observed single parameter effects	Process parameter interactions	Literature
µ_average_	Higher at increased pH, and *p*O_2_; quadratic *p*CO_2_ effects	pH × *p*CO_2_	Similar single effects [13, 15, 18, 25]
*q*_p_	Higher at increased pH, and *p*O_2_; quadratic *p*CO_2_ effects	pH × *p*CO_2_	Similar single effects [15, 20]; Contrary single effects [13, 25]
*q*_AA_	Higher uptake rates and lower production rates of several amino acids at increased pH and *p*O_2_; contrary effects of *p*CO_2_	pH × *p*CO_2_/pH × *p*O_2_	Similar single effects of process pH [13]
mAb acidic charge variant (ACV 2)	Higher at increased process pH	pH × *p*CO_2_	No literature available since variant identity is unknown
mAb sialylation (bG1FSA_2)	Quadratic pH effects	pH × *p*CO_2_	Similar interaction effect [19]

Key responses with process parameter interaction effects: average specific cell growth (*µ*_average_), specific productivity (*q*_p_), specific amino acid consumption/production rates (*q*_AA_), acidic charge variant 2 and mAb sialylation variant bG1FSA_2. Effects are only valid inside of the experimental design space. Contradictions to/in literature may derive from cell line specific effects. More detailed information is presented in the corresponding paragraphs of the “Results and discussion” section

## Conclusions

The goal of this contribution was to assess the interactions of scale dependent process parameters and their independent effects in a multivariate manner. Only, the decoupled control of process pH, *p*O_2_ and *p*CO_2_, allowed us to execute a design of experiments to investigate the interactions and independent influences of these parameters on CHO cell physiology, process performance and critical product quality attributes.

Concerning cell specific growth, glucose consumption, lactate production, amino acid metabolism and specific productivity process pH seemed to provoke the strongest effects. Variations of *p*O_2_ and *p*CO_2_ exerted influence on cell growth as well as on specific productivity, whereby we found a positive correlation for dissolved oxygen and mostly quadratic interactions for *p*CO_2_ with an optimum at around 10 % (90 mmHg). Amino acid metabolism was mainly affected by pH, but the gathered data revealed additional interactions and single effects of *p*O_2_ and *p*CO_2_.

Besides process performance, final product quality is of upmost importance for pharmaceutical bioprocesses. Therefore, critical quality attributes (CQAs), such as charge and size heterogeneity as well as *N*-glycosylation pattern were investigated. Concerning mAb aggregation and fragmentation no correlations with process pH, *p*O_2_ or *p*CO_2_ could be obtained. Significant correlations between process pH or *p*CO_2_ with mAb charge modifications as asparagine deamidation and aspartate isomerization could be derived from data out of cation exchange chromatography. The effect of process parameters on *N*-glycosylation heterogeneity is reported contradictorily in the literature and seems to be strongly dependent from the specific cell line, product and further cultivation conditions. In our study, positive correlations between antibody galactosylation, afucosylation and sialylation were found. Furthermore, highest sialylation levels could mostly be detected at pH 7.2 and highest mannose variants (Man8 and Man6) could only be observed at two distinct pH set points.

In the end, novel interactions that could be derived are pH and *p*CO_2_ interaction effects on specific cell growth (*µ*_max_, *µ*_average_) and specific productivity (*q*_P_). Moreover, several interaction effects of *p*O_2_ and *p*CO_2_ with pH on amino acid metabolism, as well as *p*CO_2_ and pH interactions on mAb charge variants and *N*-glycosylation variants were identified.

The presented results demonstrate the necessity to consider process parameter interactions on cell physiology, overall process performance and product quality. In large-scale processes, heterogeneities and gradients of process pH, *p*O_2_ and *p*CO_2_ can occur, exposing cells dynamically to changing environments. Therefore, not only the single parameter influences but also their interaction effects vary inside of large-scale processes. Especially process pH and *p*CO_2_ are usually coupled in cell culture processes, whereby zones with high pH, as they can appear due to base addition from top, simultaneously lead to low *p*CO_2_ values, whereas *p*CO_2_ accumulation can lead to zones with lower pH values, respectively. Based on our results, pH variations that might occur due to CO_2_ accumulation or base addition in large-scale are most probably the dominant factor concerning process parameter induced scale-up effects. CO_2_ accumulation in large-scale can furthermore reduce specific cell growth, specific productivity and affect amino acid metabolism. In our experiments, *p*O_2_ had the lowest effects on cell physiology and product quality. Therefore, temporary *p*O_2_ gradients that might occur in large-scale most probably only exert minor effects on process performance and CQAs. Based on the results of this study the consideration of process parameter interactions is recommended for mechanistic and hybrid modeling approaches as well as scale-up tasks.

## Electronic supplementary material

Below is the link to the electronic supplementary material.

Supplementary material 1 (DOC 125 kb)

## References

[1] Spadiut O, Capone S, Krainer F, Glieder A, Herwig C (2014). Microbials for the production of monoclonal antibodies and antibody fragments. Trends Biotechnol.

[2] Kim JY, Kim YG, Lee GM (2012). CHO cells in biotechnology for production of recombinant proteins: current state and further potential. Appl Microbiol Biotechnol.

[3] Hernandez R (2015). Top trends in biopharmaceutical manufacturing: 2015. Pharm Technol.

[4] Omasa T, Onitsuka M, Kim WD (2010). Cell engineering and cultivation of chinese hamster ovary (CHO) cells. Curr Pharm Biotechnol.

[5] Jayapal KP, Wlaschin KF, Hu W-S, Yap MGS (2007). Recombinant protein therapeutics from CHO cells-20 years and counting. Chem Eng Prog.

[6] International Conference of Harmonisation, ICH Harmonised Tripartite Guideline: Q8(R2) Pharmaceutical Development. (2009). http://www.ich.org/fileadmin/Public_Web_Site/ICH_Products/Guidelines/Quality/Q8_R1/Step4/Q8_R2_Guideline.pdf. Accessed 1 May 2016

[7] von Stosch M, Davy S, Francois K, Galvanauskas V, Hamelink JM, Luebbert A, Mayer M, Oliveira R, O’Kennedy R, Rice P, Glassey J (2014). Hybrid modeling for quality by design and PAT-benefits and challenges of applications in biopharmaceutical industry. Biotechnol J.

[8] Lara AR, Galindo E, Ramirez OT, Palomares LA (2006). Living with heterogeneities in bioreactors: understanding the effects of environmental gradients on cells. Mol Biotechnol.

[9] Nienow AW (2006). Reactor engineering in large scale animal cell culture. Cytotechnology.

[10] Xing Z, Kenty BM, Li ZJ, Lee SS (2009). Scale-up analysis for a CHO cell culture process in large-scale bioreactors. Biotechnol Bioeng.

[11] Rea JC, Wang YJ, Moreno TG, Parikh R, Lou Y, Farnan D (2012) Monoclonal antibody development and physicochemical characterization by high performance ion exchange chromatography. In: Agbo EC (ed) Innovations in biotechnology. InTech, pp 439–464. ISBN: 978-953-51-0096-6, http://www.intechopen.com/books/innovations-in-biotechnology/monoclonal-antibody-development-and-physicochemical-characterization-by-high-performance-ion-exchang

[12] Cromwell ME (2006) Formulation, filling and packaging. In: Ozturk SS, Hu W-S (eds) Cell culture technology for pharmaceutical and cell-based therapies, chap 14. Taylor & Francis Group, London, pp 483–522

[13] Trummer E, Fauland K, Seidinger S, Schriebl K, Lattenmayer C, Kunert R, Vorauer-Uhl K, Weik R, Borth N, Katinger H, Muller D (2006). Process parameter shifting: part I. Effect of DOT, pH, and temperature on the performance of Epo-Fc expressing CHO cells cultivated in controlled batch bioreactors. Biotechnol Bioeng.

[14] Kishishita S, Nishikawa T, Shinoda Y, Nagashima H, Okamoto H, Takuma S, Aoyagi H (2015). Effect of temperature shift on levels of acidic charge variants in IgG monoclonal antibodies in Chinese hamster ovary cell culture. J Biosci Bioeng.

[15] Link T, Backstrom M, Graham R, Essers R, Zorner K, Gatgens J, Burchell J, Taylor-Papadimitriou J, Hansson GC, Noll T (2004). Bioprocess development for the production of a recombinant MUC1 fusion protein expressed by CHO-K1 cells in protein-free medium. J Biotechnol.

[16] Li F, Vijayasankaran N, Shen AY, Kiss R, Amanullah A (2010). Cell culture processes for monoclonal antibody production. mAbs.

[17] Ivarsson M, Villiger TK, Morbidelli M, Soos M (2014). Evaluating the impact of cell culture process parameters on monoclonal antibody *N*-glycosylation. J Biotechnol.

[18] Dezengotita VM, Kimura R, Miller WM (1998). Effects of CO_2_ and osmolality on hybridoma cells: growth, metabolism and monoclonal antibody production. Cytotechnology.

[19] Zanghi JA, Schmelzer AE, Mendoza TP, Knop RH, Miller WM (1999). Bicarbonate concentration and osmolality are key determinants in the inhibition of CHO cell polysialylation under elevated pCO(2) or pH. Biotechnol Bioeng.

[20] Gray DR, Chen S, Howarth W, Inlow D, Maiorella BL (1996). CO(2) in large-scale and high-density CHO cell perfusion culture. Cytotechnology.

[21] Ivarsson M, Noh H, Morbidelli M, Soos M (2015). Insights into pH-induced metabolic switch by flux balance analysis. Biotechnol Prog.

[22] Goudar CT, Biener R, Konstantinov KB, Piret JM (2009). Error propagation from prime variables into specific rates and metabolic fluxes for mammalian cells in perfusion culture. Biotechnol Prog.

[23] Frahm B, Blank HC, Cornand P, Oelssner W, Guth U, Lane P, Munack A, Johannsen K, Portner R (2002). Determination of dissolved CO(2) concentration and CO(2) production rate of mammalian cell suspension culture based on off-gas measurement. J Biotechnol.

[24] Klein T, Heinzel N, Kroll P, Brunner M, Herwig C, Neutsch L (2015). Quantification of cell lysis during CHO bioprocesses: impact on cell count, growth kinetics and productivity. J Biotechnol.

[25] Yoon SK, Choi SL, Song JY, Lee GM (2005). Effect of culture pH on erythropoietin production by Chinese hamster ovary cells grown in suspension at 32.5 and 37.0 °C. Biotechnol Bioeng.

[26] Gódia F, Cairó JJ, Ozturk SS, Hu W-S (2006). Cell metabolism. Cell culture technology for pharamceutical and cell-based therapies.

[27] Carrillo-Cocom LM, Genel-Rey T, Araiz-Hernandez D, Lopez-Pacheco F, Lopez-Meza J, Rocha-Pizana MR, Ramirez-Medrano A, Alvarez MM (2015). Amino acid consumption in naive and recombinant CHO cell cultures: producers of a monoclonal antibody. Cytotechnology.

[28] Li J, Wong CL, Vijayasankaran N, Hudson T, Amanullah A (2012). Feeding lactate for CHO cell culture processes: impact on culture metabolism and performance. Biotechnol Bioeng.

[29] Zagari F, Jordan M, Stettler M, Broly H, Wurm FM (2013). Lactate metabolism shift in CHO cell culture: the role of mitochondrial oxidative activity. New Biotechnol.

[30] Wahrheit J, Nicolae A, Heinzle E (2014). Dynamics of growth and metabolism controlled by glutamine availability in Chinese hamster ovary cells. Appl Microbiol Biotechnol.

[31] Kim HS, Lee GM (2007). Differences in optimal pH and temperature for cell growth and antibody production between two Chinese hamster ovary clones derived from the same parental clone. J Microbiol Biotechnol.

[32] Lu C, Liu D, Liu H, Motchnik P (2014). Characterization of monoclonal antibody size variants containing extra light chains. mAbs.

[33] Jing Y, Borys M, Nayak S, Egan S, Qian Y, Pan S-H, Li ZJ (2012). Identification of cell culture conditions to control protein aggregation of IgG fusion proteins expressed in Chinese hamster ovary cells. Process Biochem.

[34] Liu H, Gaza-Bulseco G, Faldu D, Chumsae C, Sun J (2008). Heterogeneity of monoclonal antibodies. J Pharm Sci.

[35] Franco R, Daniela G, Fabrizio M, Ilaria G, Detlev H (1999). Influence of osmolarity and pH increase to achieve a reduction of monoclonal antibodies aggregates in a production process. Cytotechnology.

[36] Usami A, Ohtsu A, Takahama S, Fujii T (1996). The effect of pH, hydrogen peroxide and temperature on the stability of human monoclonal antibody. J Pharm Biomed Anal.

[37] Gomes JM, Hiller GW (2008) Use of low temperature and/or low ph in cell culture. US 2008/0269132 A1

[38] Terashima I, Koga A, Nagai H (2007). Identification of deamidation and isomerization sites on pharmaceutical recombinant antibody using H218O. Anal Biochem.

[39] Zhong X, Wright JF (2013). Biological insights into therapeutic protein modifications throughout trafficking and their biopharmaceutical applications. Int J Cell Biol.

[40] Liu H, Ponniah G, Zhang HM, Nowak C, Neill A, Gonzalez-Lopez N, Patel R, Cheng G, Kita AZ, Andrien B (2014). In vitro and in vivo modifications of recombinant and human IgG antibodies. mAbs.

[41] Zheng JY, Janis LJ (2006). Influence of pH, buffer species, and storage temperature on physicochemical stability of a humanized monoclonal antibody LA298. Int J Pharm.

[42] Yi L, Beckley N, Gikanga B, Zhang J, Wang YJ, Chih HW, Sharma VK (2013). Isomerization of Asp–Asp motif in model peptides and a monoclonal antibody Fab fragment. J Pharm Sci.

[43] Cai B, Pan H, Flynn GC (2011). C-terminal lysine processing of human immunoglobulin G2 heavy chain in vivo. Biotechnol Bioeng.

[44] Dick LW, Qiu D, Mahon D, Adamo M, Cheng KC (2008). C-terminal lysine variants in fully human monoclonal antibodies: investigation of test methods and possible causes. Biotechnol Bioeng.

[45] Harris RJ (1995). Processing of C-terminal lysine and arginine residues of proteins isolated from mammalian cell culture. J Chromatogr A.

[46] Agarabi CD, Schiel JE, Lute SC, Chavez BK, Boyne MT, Brorson KA, Khan M, Read EK (2015). Bioreactor process parameter screening utilizing a Plackett–Burman design for a model monoclonal antibody. J Pharm Sci.

[47] Raju TS, Jordan RE (2012). Galactosylation variations in marketed therapeutic antibodies. mAbs.

[48] Hossler P, Khattak SF, Li ZJ (2009). Optimal and consistent protein glycosylation in mammalian cell culture. Glycobiology.

[49] Kimura R, Miller WM (1997). Glycosylation of CHO-derived recombinant tPA produced under elevated *p*CO_2_. Biotechnol Prog.

