# Supplementary material for: Investigation of the interactions of critical scale-up parameters (pH, pO2 and pCO2) on CHO batch performance and critical quality attributes
Source: Bioprocess Biosyst Eng. 2016 Oct 17;40(2):251–63. doi: 10.1007/s00449-016-1693-7 (PMC5274649; doi:10.1007/s00449-016-1693-7)
Supplement: Supplementary file 1 — Supplementary material 1 (DOC 125 kb) [file 449_2016_1693_MOESM1_ESM.doc]

**Supplementary materials to the manuscript:**

**Investigation of the interactions of critical scale-up parameters (pH, pO_2_ and pCO_2_) on CHO batch performance and critical quality attributes**

**Matthias Brunner, Jens Fricke, Paul Kroll and Christoph Herwig**

Research Area of Biochemical Engineering, Vienna University of Technology, Vienna, Austria;

CD Laboratory on Mechanistic and Physiological Methods for improved Bioprocesses, Vienna University of Technology, Gumpendorferstrasse 1a/166, 1060 Vienna, Austria

**Correspondence:** Univ. Prof. Dr. Christoph Herwig, Department of Biochemical Engineering, Vienna University of Technology, Gumpendorferstrasse 1a 166/4, 1060 Vienna, Austria

**E-mail**: [christoph.herwig@tuwien.ac.at](mailto:christoph.herwig@tuwien.ac.at)

Specific data points of the gathered PLS-models

Table 1 Product quality data of the PLS-models for all batch experiments

| Exp No | bG1SA_2 [%] | ACV 2 [%] | ACV 1 [%] | BCV 1 [%] | BCV 4 [%] | BCV 2 [%] | BCV 3 [%] | sum of basic variants [%] |
| --- | --- | --- | --- | --- | --- | --- | --- | --- |
| 1 | 0.144 | 5.771 | 8.65 | 5.200 | 0.533 | 2.768 | 1.463 | 10.579 |
| 2 | 0.171 | 8.914 | 8.546 | 4.853 | 0.431 | 3.21 | 1.058 | 10.191 |
| 3 | 0.184 | 5.804 | 11.336 | 7.248 | 0.611 | 3.08 | 1.454 | 12.922 |
| 4 | 0.100 | 8.043 | 8.769 | 4.74 | 0.44 | 3.29 | 1.069 | 10.134 |
| 5 | 0.119 | 5.778 | 9.942 | 6.705 | 0.469 | 3.12 | 1.544 | 12.369 |
| 6 | 0.192 | 9.024 | 8.759 | 4.529 | 0.44 | 3.65 | 1.03 | 10.336 |
| 7 | 0.141 | 6.412 | 13.083 | 7.11 | 0.581 | 2.91 | 1.225 | 12.362 |
| 8 | - | - | - | - | - | - | - | - |
| 9 | 0.121 | 7.459 | 8.714 | 4.987 | 0.505 | 3.55 | 1.201 | 10.86 |
| 10 | 0.096 | 7.101 | 7.676 | 4.467 | 0.443 | 3.42 | 1.255 | 10.296 |
| 11 | 0.095 | 7.075 | 9.33 | 4.434 | 0.48 | 3.37 | 1.154 | 9.957 |
| 12 | 0.151 | 8.873 | 7.881 | 3.708 | 0.442 | 3.33 | 1.057 | 9.237 |
| 13 | 0.115 | 5.697 | 8.924 | 5.89 | 0.541 | 2.92 | 1.53 | 11.054 |
| 14 | 0.123 | 6.89 | 6.975 | 4.575 | 0.423 | 3.45 | 1.359 | 10.489 |
| 15 | 0.096 | 5.02 | 10.544 | 4.641 | 0.419 | 3.03 | 0.927 | 10.351 |
| 16 | 0.108 | 5.157 | 8.057 | 4.657 | 0.482 | 2.74 | 1.128 | 10.107 |
| 17 | 0.104 | 6.535 | 7.765 | 4.932 | 0.484 | 3.23 | 1.226 | 10.563 |
| 18 | 0.101 | 7.03 | 9.036 | 4.216 | 0.483 | 3.36 | 1.182 | 9.88 |
| 19 | 0.125 | 6.35 | 7.77 | 4.558 | 0.483 | 3.44 | 1.318 | 10.433 |

Table 2 Specific cell growth, lactate production, glucose consumption and specific productivity of the PLS-models for all batch experiments

| Exp No | µaverage [1/h] | µmax [1/h] | qlac [mmol/(10E09 cells*h)] | qgluc [mmol/(10E09 cells*h)] | qp [mg/(10E09 cells*h)] |
| --- | --- | --- | --- | --- | --- |
| 1 | 0.020 | 0.025 | 0.069 | -0.090 | 0.823 |
| 2 | 0.032 | 0.035 | 0.293 | -0.213 | 1.139 |
| 3 | 0.022 | 0.027 | 0.081 | -0.128 | 0.879 |
| 4 | 0.027 | 0.031 | 0.209 | -0.232 | 0.937 |
| 5 | 0.024 | 0.029 | 0.084 | -0.104 | 0.948 |
| 6 | 0.034 | 0.039 | 0.262 | -0.212 | 1.09 |
| 7 | 0.023 | 0.029 | 0.060 | -0.083 | 1.026 |
| 8 | - | 0.037 | - | - | - |
| 9 | 0.026 | 0.034 | 0.127 | -0.101 | 1.112 |
| 10 | 0.025 | 0.035 | 0.149 | -0.118 | 1.045 |
| 11 | 0.028 | 0.037 | 0.205 | -0.135 | 1.020 |
| 12 | 0.031 | 0.037 | 0.219 | -0.204 | 1.146 |
| 13 | 0.023 | 0.031 | 0.0727 | -0.109 | 0.904 |
| 14 | 0.027 | 0.036 | 0.132 | -0.157 | 1.045 |
| 15 | 0.025 | 0.034 | 0.136 | -0.185 | 0.821 |
| 16 | 0.021 | 0.033 | 0.169 | -0.121 | 0.912 |
| 17 | 0.027 | 0.037 | 0.189 | -0.148 | 1.075 |
| 18 | 0.024 | 0.035 | 0.166 | -0.123 | 1.031 |
| 19 | 0.028 | 0.035 | 0.154 | -0.141 | 1.102 |

Table 3 Specific amino acid consumption/production rates (Asp, Ser, His and Arg) of the PLS-models for all batch experiments. Several measurement points are missing due to missing samples

| Exp No | qAsp [mg/(10E09 cells*h)] | qSer [mg/(10E09 cells*h)] | qHis [mg/(10E09 cells*h)] | qArg [mg/(10E09 cells*h)] |
| --- | --- | --- | --- | --- |
| 1 | 0.547 | -1.017 | -0.127 | -0.204 |
| 2 | - | - | - | - |
| 3 | 0.363 | -1.640 | -0.193 | - |
| 4 | -0.087 | -1.674 | -0.177 | -0.728 |
| 5 | 0.405 | -1.153 | -0.182 | -0.575 |
| 6 | 0.106 | -1.632 | -0.247 | -0.693 |
| 7 | 0.175 | -1.453 | -0.209 | -0.652 |
| 8 | - | - | - | - |
| 9 | - | -1.039 | -0.159 | -0.539 |
| 10 | - | -1.175 | -0.195 | -0.547 |
| 11 | 0.002 | -1.269 | -0.179 | -0.427 |
| 12 | -0.212 | -1.513 | -0.204 | -0.628 |
| 13 | - | -1.086 | -0.142 | -0.259 |
| 14 | - | - | - | - |
| 15 | -0.024 | -1.428 | -0.187 | -0.601 |
| 16 | -0.089 | -1.226 | -0.154 | -0.370 |
| 17 | -0.163 | -1.368 | -0.170 | -0.679 |
| 18 | - | -1.253 | -0.164 | -0.520 |
| 19 | -0.1 | -1.369 | -0.188 | -0.514 |

Table 4 Specific amino acid consumption/production rates (Asp, Ser, His and Arg) of the PLS-models for all batch experiments. Several measurement points are missing due to missing samples

| Exp No | qGly [mg/(10E09 cells*h)] | qCys [mg/(10E09 cells*h)] | qVal [mg/(10E09 cells*h)] | qILeu [mg/(10E09 cells*h)] | qGlu [mg/(10E09 cells*h)] |
| --- | --- | --- | --- | --- | --- |
| 1 | 0.479 | 0.720 | -0.362 | -0.267 | 0.196 |
| 2 | 0.302 | 0.304 | -1.062 | -1.177 | - |
| 3 | 0.499 | 0.680 | -0.592 | -0.623 | 0.045 |
| 4 | 0.408 | 0.434 | -0.705 | -0.484 | 0.025 |
| 5 | 0.218 | 0.256 | -0.580 | -0.589 | 0.150 |
| 6 | 0.326 | 0.440 | -0.825 | -0.851 | 0.176 |
| 7 | 0.335 | 0.320 | -0.651 | -0.673 | 0.056 |
| 8 | - | - | - | - | - |
| 9 | 0.357 | 0.378 | -0.508 | -0.527 | - |
| 10 | 0.394 | 0.376 | -0.508 | -0.609 | - |
| 11 | 0.367 | 0.496 | -0.487 | -0.465 | 0.117 |
| 12 | 0.320 | 0.442 | -0.641 | -0.674 | 0.119 |
| 13 | 0.366 | 0.548 | -0.406 | -0.349 | - |
| 14 | - | - | - | - | - |
| 15 | 0.397 | 0.541 | -0.590 | -0.611 | 0.088 |
| 16 | 0.344 | 0.474 | -0.409 | -0.384 | 0.050 |
| 17 | 0.259 | 0.217 | -0.526 | -0.553 | 0.048 |
| 18 | 0.408 | 0.470 | -0.571 | -0.599 | 0.058 |
| 19 | 0.358 | 0.502 | -0.564 | -0.557 | 0.068 |
